# Proteomic Signatures Reveal Differences in Stress Response, Antioxidant Defense and Proteasomal Activity in Fertile Men with High Seminal ROS Levels

**DOI:** 10.3390/ijms20010203

**Published:** 2019-01-08

**Authors:** Tânia R. Dias, Luna Samanta, Ashok Agarwal, Peter N. Pushparaj, Manesh Kumar Panner Selvam, Rakesh Sharma

**Affiliations:** 1 American Center for Reproductive Medicine, Cleveland Clinic, Cleveland, OH 44195, USA; taniadias89@gmail.com (T.R.D.); lsamanta@ravenshawuniversity.ac.in (L.S.); manesh.balu@gmail.com (M.K.P.S.); sharmar@ccf.org (R.S.); 2 Universidade da Beira Interior, 6201-001 Covilhã, Portugal; 3 Department of Microscopy, Laboratory of Cell Biology, Institute of Biomedical Sciences Abel Salazar and Unit for Multidisciplinary Research in Biomedicine, University of Porto, 4050-313 Porto, Portugal; 4 Redox Biology Laboratory, Department of Zoology, School of Life Sciences, Ravenshaw University, Cuttack 753003, Odisha, India; 5 Center of Excellence in Genomic Medicine Research, Jeddah 21589, Saudi Arabia; peter.n.pushparaj@gmail.com

**Keywords:** seminal plasma, spermatozoa, reactive oxygen species, antioxidants, chemiluminescence, proteomics, bioinformatics, differentially expressed proteins, Western blot

## Abstract

Elevated levels of reactive oxygen species (ROS) are a major cause of male infertility. However, some men with high seminal ROS levels are still fertile. The main objective of this study was to understand the molecular mechanism(s) responsible for the preservation of fertility in those men. Semen samples from fertile men were divided into two groups: control (*n* = 10, ROS < 102.2 RLU/s/10^6^ sperm) and ROS+ (*n* = 10, ROS > 102.2 RLU/s/10^6^ sperm). Proteomic analysis of seminal plasma and spermatozoa was used to identify the differentially expressed proteins (DEPs) between the experimental groups, from which some proteins were validated by Western blot (WB). A total of 44 and 371 DEPs were identified between the study groups in the seminal plasma and spermatozoa, respectively. The identified DEPs were primarily involved in oxidoreductase, endopeptidase inhibitor, and antioxidant activities. We validated by WB the underexpression of NADH:ubiquinone oxidoreductase core subunit S1 (*p* = 0.01), as well as the overexpression of superoxide dismutase 1 (*p* = 0.03) and peroxiredoxin 4 (*p* = 0.04) in spermatozoa of ROS+ group. Our data suggest that fertile men with high ROS levels possess an effective antioxidant defense system that protects sperm proteins, as well as an active proteasomal system for degradation of defective proteins.

## 1. Introduction

A common end to numerous pathways that lead to defective sperm function is the increase in reactive oxygen species (ROS) levels in semen [1,2]. Physiological levels of ROS in the semen are essential for an optimal sperm function and fertilization, as they participate in motility acquisition, capacitation, and acrosome reaction [3,4]. However, when the rate of ROS generation exceeds the cells’ antioxidant defense capacity, it leads to oxidative stress (OS), which may damage sperm DNA, lipids and proteins, thus compromising sperm fertilizing potential [3]. Spermatozoa possess a limited intrinsic antioxidant machinery that make them dependent on seminal plasma defense system [5]. This characteristic increases the interest regarding the clinical utility of seminal OS testing in infertility clinics [6,7].

Besides routine semen analysis, advanced sperm function tests for the assessment of ROS levels, total antioxidant capacity, sperm DNA fragmentation and compaction, as well as genetic testing are currently used for the evaluation of male fertility status [8]. Nevertheless, these tests are unable to establish the etiology of infertility, leading to the classification of many cases as idiopathic [8]. Even though the chances of conception are increased by assisted reproductive technology (ART) in these patients, the genomic stability of the embryo is not guaranteed [9]. OS-induced sperm DNA damage is the cause of infertility in many men [10,11]. In fact, many infertile men with high ROS levels show sperm DNA fragmentation and poor chromatin packaging [9]. This is associated with lower fertilization and pregnancy rates in ART, impaired embryo development and quality; and increased risk of spontaneous abortions, birth defects and childhood diseases such as cancer [10,11,12]. In recent years, proteomic analysis of the semen has helped in understanding the biological pathways associated with male infertility [13]. Our group has extensively studied the proteomic profile of both seminal plasma and spermatozoa from men with different fertility-related conditions, giving attention to ROS levels [14,15,16]. During these investigations, we noticed that some healthy men who presented high ROS levels in their ejaculates were able to father children. The cutoff to classify a semen sample as containing high ROS levels was 102.2 relative light units per second per million of spermatozoa (RLU/s/10^6^ sperm), as previously established [17]. Therefore, we decided to explore the molecular mechanisms by which these men preserve their fertility. The goal of this study was to compare the proteome of seminal plasma and spermatozoa from fertile men with high ROS levels with that of fertile men with physiological ROS levels. We aimed to identify possible alterations in the expression levels of key antioxidant proteins, as well as the underlying pathways responsible for the protection of spermatozoa from ROS attack.

## 2. Results

### 2.1. Semen Analysis and ROS Levels

All samples in both the groups were normozoospermic according to World Health Organization (WHO) 2010 criteria [18] (Table 1). There were no significant differences in semen parameters between the control and the ROS+ groups. ROS levels were higher (*p* = 0.0001) in ROS+ group compared to the control group (Table 1).

### 2.2. Global Proteomic Profile of Seminal Plasma and Spermatozoa

**Figure 1:**
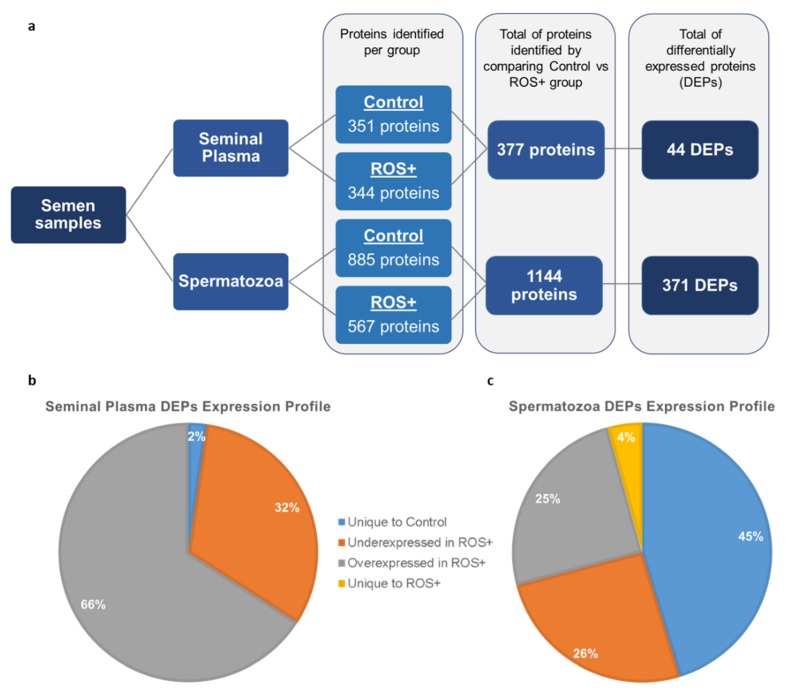
Schematic representation of the results obtained by proteomic analysis: (**a**) number of proteins identified in the seminal plasma and spermatozoa of fertile men (control) and men with high levels of reactive oxygen species (ROS+), as well as the number of differentially expressed proteins (DEPs) between the experimental groups; (**b**) expression profile of seminal plasma DEPs; and (**c**) expression profile of spermatozoa DEPs.

Proteomic analysis of seminal plasma resulted in the identification of 351 proteins in the control group and 344 proteins in ROS+ group. From a total of 377 proteins in both groups, 44 were differentially expressed proteins (DEPs) (Figure 1a). One of the seminal plasma DEPs was unique to the control group (2%), while 29 were overexpressed (66%), and 14 underexpressed (32%) in ROS+ group (Figure 1b).

In spermatozoa, 885 and 567 proteins were identified in the control and ROS+ groups, respectively. A total of 1144 proteins where identified after the comparison between both groups, from which 371 proteins were differentially expressed (Figure 1a). The majority (45%) of the spermatozoa DEPs were unique to the control group (168 proteins), while only 16 proteins were unique to the ROS+ group (4%). Besides, 95 DEPs were underexpressed (26%) and 92 overexpressed (25%) in ROS+ group (Figure 1c).

### 2.3. Functional Annotations and Pathway Analysis

**Figure 2:**
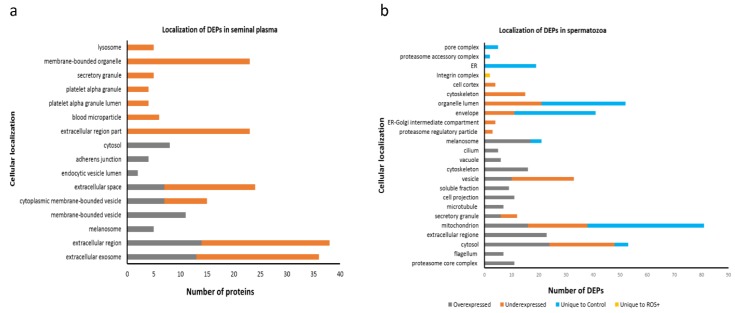
Localization of differentially expressed proteins (DEPs) in: (**a**) seminal plasma; and (**b**) spermatozoa. The number of DEPs that were overexpressed (grey), underexpressed (orange), unique to control (blue), and unique to ROS+ (yellow) are shown for seminal plasma and spermatozoa.

Protein annotations revealed that the DEPs identified in seminal plasma belong to exosomes, different vesicles, secretory granules, and extracellular proteins (Figure 2a). However, membrane-bound organelle proteins were also detected in seminal plasma (Figure 2a). In spermatozoa, the identified DEPs belong to various subcellular locations such as mitochondria and flagellum cytoskeleton (Figure 2b).

Functional enrichment analysis of seminal plasma DEPs using STRING online software showed the biological processes and molecular functions in which they were involved. According to the biological processes, 4 DEPs were involved in acute phase response, 6 in protein folding and 18 in regulation of biological quality. Regarding the molecular functions, 4 DEPs were associated with antioxidant activity and 7 with endopeptidase inhibitor activity. Haptoglobin (HP), peroxiredoxin 4 (PRDX4) and S100 calcium-binding protein A9 (S100A9) were the main proteins involved in antioxidant activity, while serpin B6 (SERPINB6) and complement C3 (C3) were among the proteins involved in endopeptidase inhibitor activity. According to the Ingenuity Pathway Analysis (IPA) semenogelins I (SEMG1) and II (SEMG2) were in the top list of downregulated proteins in seminal plasma with a higher fold change between the groups. On the other hand, HP and C3 were among the top list of upregulated proteins with a higher fold change in ROS+ relative to control group. These two proteins were also classified as positive acute phase response proteins, which was one of the toxicity functions identified by the Tox lists tool (Supplementary Figure S1a). PRDX4 and S100A9 were associated with OS as identified by the IPA Tox lists tool (Supplementary Figure S1a). These seven DEPs were selected for validation by Western blot (WB) and compared with the results obtained by the proteomic results (Table 2).

In spermatozoa, the functional enrichment analysis of Search Tool for the Retrieval of Interacting Genes/Proteins (STRING) software showed that, among the biological processes, 76 proteins were associated with response to stress, 19 with protein folding, 37 were involved in oxidation-reduction processes, and 42 in the regulation of response to stress. Regarding the molecular functions, 11 proteins presented antioxidant activity, including superoxide dismutase 1 (SOD1), PRDX4, thioredoxin reductase 1 and 2 (TXNRD1 and TXNRD2). Moreover, 28 proteins were associated with oxidoreductase activity, comprising NADH-ubiquinone oxidoreductase core subunit S1 (NDUFS1), TXNRD2, SOD1 and PRDX4. After performing the IPA analysis, similar results were observed by the IPA Tox lists tool (Supplementary Figure S1b). PRDX4, SOD1 and TXNRD2 were associated with OS, while NDUFS1 and TXNRD2 were related to mitochondrial dysfunction. Besides, SOD1 and TXNRD1 were also associated with NRF2-mediated OS response. 5 proteins were selected for validation by WB and compared with the results obtained by the proteomic analysis (Table 3).

### 2.4. Upstream Regulators

Using the upstream analysis tool of IPA, several cytokines were predicted to be responsible for the altered expression levels of seminal plasma proteins in the dataset. Interleukin-1 alpha and beta (IL1A and IL1B), interleukin-6 (IL6), Interleukin-22 (IL22), and tumor necrosis factor (TNF) were predicted to be activated, explaining the overexpression of DEPs such as S100A9, C3 and HP. They may also be responsible for the underexpression of prostate-specific antigen (KLK3), lipoprotein lipase (LPL) and chaperone heat shock protein HSP 90-beta (HSP90AB1) (Supplementary Figure S2).

In spermatozoa, two upstream regulators were predicted to be activated in this dataset: nuclear factor erythroid 2-related factor 2 (NFE2L2) and TNF. The transcription regulator NFE2L2 was shown to regulate the overexpression of proteins involved in oxidation-reduction processes, such as SOD1, SOD2 and 6-phosphogluconate dehydrogenase, decarboxylating (PGD) (Supplementary Figure S3). Its activation may also explain the overexpression of some proteasomes (PSMB2 and PSMB5). The cytokine TNF was also predicted to be activated and regulate the overexpression of SOD2, fibronectin (FN1), ion-binding proteins (GPD2, HSPG2, LCN2), as well as the underexpression of prohibitin (PHB) (Figure S3).

### 2.5. Western Blot

**Figure 3:**
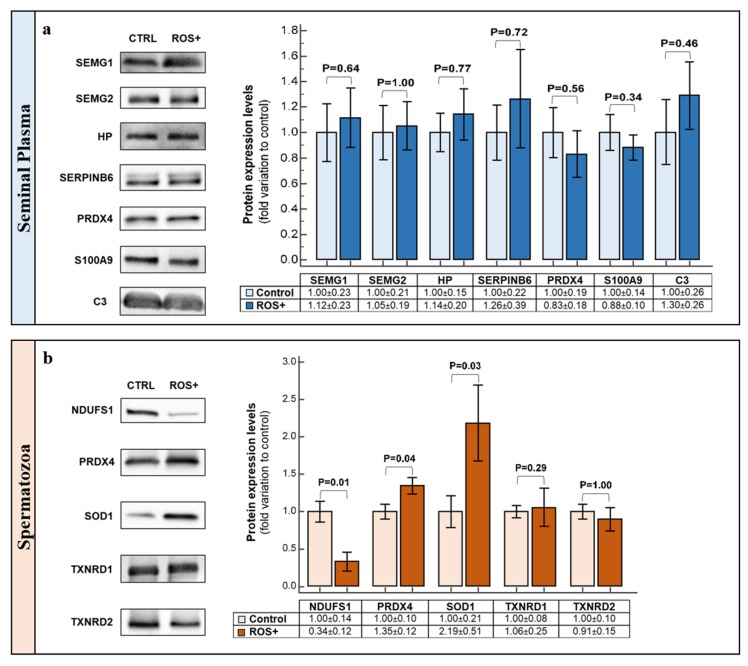
Graphical representation of Western blot results and respective representative blots for: (**a**) seminal plasma; and (**b**) spermatozoa proteins. Results are expressed as mean ± SEM and were considered significant for *p* < 0.05.

All the selected seminal plasma proteins (SEMG1, SEMG2, HP, SERPINB6 and PRDX4) were identified by WB, however, there were no significant alterations in their expression levels between the control and the ROS+ groups (Figure 3a).

In sperm proteins, there was a decrease in NDUFS1 (*p* = 0.01) protein expression levels in the ROS+ group relative to the control (Figure 3b). An overexpression of PRDX4 (*p* = 0.04) and SOD1 (*p* = 0.03) was observed in ROS+ group when compared to the control group. There were no significant alterations in the protein expression of TXNRD1 and TXNRD2 (Figure 3b).

## 3. Discussion

High seminal ROS levels have been widely debated as a major cause of male infertility [19,20]. Nevertheless, the role of ROS at physiological concentrations in regulation of sperm function cannot be ignored [3,21]. In the present study, we report a comparative proteomic analysis of seminal plasma and spermatozoa from fertile men exhibiting higher ROS levels than the pre-established reference level with respect to fertile men with basal ROS levels. This is important to gain a better insight into the role of ROS in sperm function in general and to understand sperm dysfunction under pathophysiological conditions with elevated ROS level.

In semen, the principal source of ROS are morphologically abnormal, immature spermatozoa, and leukocytes [22]. As both groups were negative for leukocytes (Endtz negative), the elevated ROS generation may be attributed to the presence of immature cells in these samples. Recently, we have reported the presence of immature cells with different proteome profile in the ejaculated semen of fertile men [23]. Therefore, the difference in the proteome profile of spermatozoa in the control and ROS+ groups may be due to the presence of comparatively more number of immature spermatozoa in the latter group. This was corroborated by our proteomic results that showed an underexpression of sperm surface protein Sp17 (SPA17) in ROS+ group. This protein is weakly expressed in spermatocytes, while a high expression was reported in early and late spermatids, which suggests that most of the ejaculated spermatozoa express SPA17 protein. This also supports its role in the sperm differentiation [24,25]. Similarly, underexpression of annexins (1–6) points towards failure of apoptosis in these samples, resulting in the increase in immature/or undifferentiated spermatozoa.

After bioinformatic analysis of the seminal plasma DEPs, we focused on SEMG1, SEMG2, SERPINB6, HP, PRDX4, S100A9 and C3. SEMGI and SEMGII are highly abundant in seminal plasma and are responsible for the formation of the characteristic gel-like coagulum after ejaculation [26]. They play an important role in protecting the spermatozoa and in the fertilization process [27]. The underexpression of SEMG1 and SEMG2 in ROS+ men was accompanied by the underexpression of KLK3, which is one of the trypsin-like serine proteases responsible for semenogelins digestion to attain semen liquefaction [28]. Moreover, an overexpression of SERPINB6 was observed in ROS+ men. This protein is a member of the serpins protein family that is involved in the regulation of trypsin-like serine proteases activity [29]. The alterations in the expression profile of these proteins resulted in normal liquefaction of semen samples in ROS+ group, an important factor for the preservation of sperm fertilizing potential.

HP, PRDX4 and S100A9 were identified as the main seminal plasma proteins involved in antioxidant activity, which were overexpressed in ROS+ samples. HP in human fluids binds to hemoglobin to inhibit its oxidative potential as a free molecule [30]. In the presence of hydrogen peroxide (H_2_O_2_), one of the main ROS in semen, hemoglobin can act as a peroxidase [31], thus generating more ROS. Overexpression of HP in the seminal plasma of ROS+ men can prevent an oxidative chain reaction. PRDX4 belongs to the family of peroxiredoxins, which are major players of the antioxidant defense system in semen. This protein was previously identified in both seminal plasma and spermatozoa of human semen samples [32]. PRDX4 contain two cysteine residues in its active site, which are major targets for ROS [33]. As ROS are neutralized after binding to PRDX4, the overexpression of this protein in the seminal plasma of ROS+ men confers higher protection against increased ROS levels.

S100A9 is a calcium- and zinc-binding protein associated with stress response [34]. It is considered a danger- or damage-associated molecular pattern (DAMP) molecule, as, in response to various stimuli, it can bind to pro-inflammatory receptors and initiate an inflammatory reaction [35]. In this particular study, the stimuli for the overexpression of this protein was the high ROS levels in semen of ROS+ men. In fact, there is a direct link between high ROS levels and inflammation [36]. A previous proteomic study also identified the overexpression of S100A9 in the seminal plasma of smoking men [37], which also reflects an environment with high ROS levels. Overexpression of S100A9 was associated with the activation of NADPH oxidase [38], which may be one of the reasons for the accumulation of ROS in semen. S100A9 pro-inflammatory activity starts with the activation of the nuclear factor-kappa B (NF-κB), which consequently induces cytokine secretion [38]. This may explain why many interleukins were predicted to be active in the seminal plasma of ROS+ men, including IL1A, IL1B, IL6, IL22, and TNF (Supplementary Figure S2). These inflammatory factors were identified as the upstream regulators of many proteins in the dataset and are implicated in the regulation of sperm fertilization processes during sperm transit through the female reproductive tract [39]. Accordingly, Tox lists showed that many positive acute phase response proteins were upregulated in ROS+ men. This may also be related to the observed overexpression of protein C3, which is a mediator of local inflammatory processes and immune responses [40]. For instance, it has been demonstrated that cytokines IL1A, IL1B, IL6 and TNF can lead to increased C3 secretion [41]. In human seminal plasma, C3 complement system is regulated by complement-inhibiting factors to protect spermatozoa from damage by chronic inflammation [42]. Although all the selected proteins were identified by WB, the results were not concordant with the proteomic data (Figure 3a).

Spermatozoa proteomic data showed 371 DEPs, from which 5 were selected for validation by WB: NDUFS1, SOD1, PRDX4, TXNRD1, and TXNRD2. NDUFS1 is one of the subunits of the mitochondrial complex I, which is the starting point of oxidative phosphorylation (OXPHOS). Complex I is responsible for NADH oxidation, thus providing electrons for the respiratory chain [43]. Mitochondrial function is crucial for sperm fertilization, not only for ATP production to obtain energy, but also for the physiological production of ROS. NDUFS1 is the largest subunit of complex I and is essential for the proper assembly of the complex required for its function [44]. The underexpression of NDUFS1 in the spermatozoa of ROS+ men may impair complex I assembly and result in its dysfunction, which is one of the most common mitochondrial dysfunctions observed in humans [44]. Moreover, subunits of complex IV (COX4I1 and COX5A) and complex V (ATP5H) were also underexpressed in ROS+ group. These alterations contribute to the higher production of ROS levels in this group. We were able to validate the underexpression of NDUFS1 by WB. Mitochondrial dysfunction in mature spermatozoa may contribute to the high ROS levels in ROS+ group. 

The preponderance for OS in spermatozoa of ROS+ group is counteracted by the increased antioxidant defense. Both cytosolic and mitochondrial superoxide dismutase (SOD1 and SOD2, respectively), mitochondrial thioredoxin reductase 2 (TXNRD2), and PRDX4 were overexpressed in spermatozoa of ROS+ group. Moreover, cytosolic thioredoxin reductase 1 (TXNRD1) was uniquely expressed in ROS+ group providing additional defense. SOD1 belongs to the superoxide dismutase family and is one of the first line of antioxidant defense enzymes against ROS attack in spermatozoa [45]. The overexpression of SOD1, which was further confirmed by the WB analysis (Figure 3b), may explain the higher antioxidant protection in spermatozoa of ROS+ men. This protein provides protection against the attack from superoxide anion radicals. SOD1 and SOD2 increased activity was predicted to be regulated by NFE2L2 and TNF, which were identified as their activated upstream regulators (Supplementary Figure S3). These transcription factors were described as important regulators of antioxidant responses [46].

PRDX4 is one of the main proteins responsible for reduction of peroxides in spermatozoa [33]. It can be found in sperm plasma membrane, acrosome, nucleus, and cytosol [33]. The binding of ROS to the active site of PRDX4 leads to the oxidation of its cysteine residues and the enzyme becomes inactive [47]. Without an active thioredoxins system, PRDX4 would remain permanently inactive in an environment with high ROS levels, thus being unable to scavenge other forms of ROS. The thioredoxin system is constituted by thioredoxins, thioredoxins reductases and NADPH [48]. Thioredoxins reductases, including TXNRD1 (cytosolic) and TXNRD2 (mitochondrial), play a key role in maintaining the cyclicity of this system; they are responsible for maintaining thioredoxins in their reduced (active) state in a NADPH-dependent manner [33]. Subsequently, thioredoxins act as electron donors for peroxiredoxins, facilitating their reduction and reactivation [47]. Based on our proteomic data, PRDX4 and TXNRD2 were overexpressed, while TXNRD1 was unique in the spermatozoa of ROS+ men. This indicates that this ROS-scavenging system is highly enhanced and responsible for the redox homeostasis in fertile men. In fact, lower levels of peroxiredoxins have been reported in the spermatozoa of infertile men [49]. Through WB, we were able to validate the overexpression of PRDX4 in ROS+ men (Figure 3b), although no differences were found for TXNRD1 and TXNRD2 between the experimental groups.

ROS can also cause oxidative modification of proteins leading to loss of structure and function or gain in undesirable function. These proteins result in structural changes by oxidative modification, and expose the hydrophobic interior of the protein, which is recognized by 20S proteasome for its effective clearance [50]. IPA pathway analysis of DEPs identified the overexpression of 11 proteasome subunits, namely, PSMA1, PSMA2, PSMA3, PSMA4, PSMA5, PSMA6, PSMA7, PSMB1, PSMB2, PSMB3, PSMB5 in ROS+ group, which indicate an efficient regulation of the protein turnover [51]. Future studies need to be done to validate the proteasomal pathway in fertile ROS+ men.

The discrepancies between the proteomic and WB results may be related to the differences in the specificity and sensitivity of the two techniques. In shotgun proteomics, liquid chromatography–tandem mass spectrometry (LC-MS/MS) data recognizes a protein when at least two peptide fragments are detected for the protein of interest. However, in WB, the detection of protein is based on the epitope against which the primary antibody is generated. As in LC-MS/MS only tryptic digestion is considered, it was easy to match the peptide sequence and identify this from the database. In the case of seminal plasma, various mucolytic and proteolytic enzymes often cleave the matrix proteins to release the spermatozoa after liquefaction. In our study, we used completely liquefied semen samples, therefore, the peptide fragments may acquire different molecular masses than the predicted ones, making the detection by WB difficult. For example, semenogelins, which are highly abundant proteins in seminal plasma, are cleaved into smaller peptides during the process of liquefaction and show multiple bands in WB. This makes the quantitation at a specific molecular weight unpractical. A limitation of this study was the small sample size due to the difficulty to enroll sufficient number of men who are fertile and positive for ROS and willing to participate in a study.

This study represents an important step towards the understanding of the molecular dynamics of sperm and seminal plasma involved in fertility preservation. We confirmed our hypothesis by demonstrating the overexpression of several antioxidant proteins in both seminal plasma and spermatozoa of proven fertile men with high ROS levels. These results indicate that in an environment of higher ROS production, some men possess the molecular machinery essential to modulate the expression of several seminal proteins to control ROS deleterious effects. Our findings suggest that the DEPs involved in proteasomal pathway and antioxidant defense may be targeted for development of new antioxidant therapies for infertile men with high seminal ROS levels.

## 4. Materials and Methods

### 4.1. Ethical Approval

This study (14-235) was conducted after approval by the Institutional Review Board (IRB) from the Cleveland Clinic.

### 4.2. Semen Analysis

A total of 20 semen samples from healthy volunteers with proven fertility were used in this study after informed written consent. The inclusion criteria were: normozoospermic men according to the WHO 2010 guidelines [18], who fathered a child in the last two years. Semen samples were collected by masturbation into a sterile container after 2–5 days of sexual abstinence and immediately incubated at 37 °C for 30 min to allow liquefaction. After complete liquefaction, the volume, pH, viscosity and color were evaluated. For hyperviscous samples, the viscosity was broken down by repeated pipetting to avoid interference of proteolytic enzymes in proteomic analysis [5]. Microscopic evaluation of the samples including sperm motility, concentration, and presence of round cells was performed using a disposable Leja counting chamber (Spectrum Technologies, Healdsburg, CA). Endtz test [52] was performed for samples with round cells >1 × 10^6^/mL and samples with leukocytospermia were excluded.

### 4.3. Measurement of Reactive Oxygen Species

The ROS levels in the semen samples were measured by a luminol-based chemiluminescence assay as previously described [53] using a Berthold luminometer (Autolumat Plus 953, Oakridge, TN, USA). ROS levels were taken into consideration to segregate the samples into: control (*n* = 10; ROS < 102.2 RLU/s/10^6^ sperm) or ROS+ (*n* = 10; ROS > 102.2 RLU/s/10^6^ sperm) groups [17].

### 4.4. Protein Extraction and Quantification

Spermatozoa were separated from the seminal plasma by centrifugation at 400× *g* for 20 min, washed 3 times in phosphate buffer saline (PBS) and finally re-suspended in radio-immunoprecipitation assay buffer (RIPA) supplemented with EDTA-free protease inhibitor cocktail (cOmplete ULTRA Tablets; Roche, Indianapolis, IN, USA) and digested overnight at 4 °C. The sperm lysates were centrifuged at 14,000× *g* for 30 min at 4 °C and the supernatant was taken for the experiments. Seminal plasma was further centrifuged at 10,000× *g* for 10 min to eliminate possible remaining cells or debris, checked under microscope for presence of spermatozoa, if any, and centrifuged again to get clear seminal plasma devoid of spermatozoa. PBS supplemented with protease inhibitor was added to seminal plasma and it was again centrifuged at 10,000× *g* for 10 min. Total protein content of both the fractions i.e., seminal plasma and spermatozoa were estimated by bicinchoninic acid method using Pierce BCA Protein Assay kit (Thermo Fisher Scientific, Waltham, MA, USA) according to the manufacturer’s instructions.

### 4.5. Quantitative Proteomic Analysis

From the 20 semen samples collected, ten were used for the quantitative proteomic analysis. Five protein samples of seminal plasma and spermatozoa were randomly selected from experimental group (control and ROS+) to maintain the biological variability. After extraction of proteins, the proteomic analysis of seminal plasma and spermatozoa fractions was carried out by LC-MS/MS. Four pooled samples were prepared: (i) spermatozoa proteins (*n* = 5) from control group; (ii) seminal plasma proteins (*n* = 5) from control group; (iii) spermatozoa proteins (*n* = 5) from ROS+ group; and (iv) seminal plasma proteins (*n* = 5) from ROS+ group. Each pool was regarded as an individual sample for the proteomic analysis. To maintain the technical variability, each of these four pooled samples were run in triplicate during LC-MS/MS analysis. Proteins were analyzed in a Finnigan LTQ-Obitrap Elite hybrid mass spectrometer system using the previously described conditions [4,54]. The resulting spectra were analyzed by the Proteome Discoverer (Thermo Fisher Scientific, Waltham, MA, USA; version 1.4.1.288) software. Database-searching algorithms from Mascot, SEQUEST and X!Tandem software were used to identify peptides/proteins from the mass spectra. The search was defined to the human protein reference database. Search results were then uploaded into the program Scaffold (Proteome Software Inc., Portland, OR, USA; version 4.0.6.1), which uses probability and statistical methods for label-free quantitation and identification of DEPs. Only protein identifications with a 99.0% probability to achieve a false discovery rate less than 1.0% and containing at least two identified peptides were considered. The abundance of each protein (very low, low, medium or high) was determined by the spectral counts. The expression profile of the DEPs between the experimental groups is based on the normalized spectral abundance factor (NSAF) ratio, which allows the identification of the proteins that are unique, underexpressed or overexpressed. The categorization of overall abundance and the identification of DEPs between the experimental groups was performed with the previously described criteria [54].

### 4.6. Bioinformatic Analysis

Publicly available bioinformatics annotation tools and databases such as GO Term Finder, GO Term Mapper, UniProt, and Software Tools for Researching Annotations of Proteins (STRAP) were used for functional annotation and enrichment analysis [55,56]. For the large list of proteins derived from proteomic study, Database for Annotation, Visualization and Integrated Discovery (DAVID) (http://david.niaid.nih.gov), and proprietary software package such as IPA from Ingenuity^®^ Systems were used to obtain consensus based, comprehensive functional context, and to conduct Tox lists and upstream analysis related to the identified DEPs. Tox lists provide a list of processes that may be affected by the altered proteomic profile, while upstream analysis tool allows the identification of the upstream regulators that may be responsible for the expression changes observed in the dataset. STRING (https://string-db.org/) was used for protein–protein interaction analysis. Based on the bioinformatic analysis, key proteins were selected for validation by WB for both seminal plasma and spermatozoa. The proteins were selected based on their involvement in ROS-related mechanisms, including in the antioxidant defense system and mitochondrial function. Besides, we focused on proteins already described in the literature as important for spermatozoa or seminal plasma functions.

### 4.7. Western Blot

The remaining 10 semen samples were used for validation of proteomic data by WB. Five protein samples from each experimental group (control and ROS+) were used individually to validate the selected proteins of seminal plasma (*n* = 5) and spermatozoa (*n* = 5). 25 µgof each spermatozoa protein sample and 50 µg of each seminal plasma protein sample were mixed with 4× Laemmli sample buffer (BioRad, Hercules, CA, USA) in a ratio 1:3 and completed up to 25 µL with PBS. Polyvinylidene difluoride (PVDF) membranes were incubated overnight (4 °C) with specific primary antibodies followed by the respective secondary antibodies at room temperature, for 90 min (Supplementary Table S1). Membranes were reacted with enhanced chemiluminescence (ECL) reagent (GE Healthcare, Marlborough, MA, USA) for 5 min and read with the ChemiDoc™ MP Imaging System (BioRad, Hercules, CA, USA) to detect the chemiluminescence signals. Densities from each band were obtained with Image Lab™ Software (BioRad, Hercules, CA, USA) according to standard methods and divided by the corresponding total protein lane density. Results were expressed as fold change relative to the control group.

### 4.8. Statistical Analysis

Semen parameters and WB results were tested for normality using the Kolmogorov–Smirnov test. As data did not present a normal distribution, results were analyzed by a non-parametric Mann–Whitney test for independent samples, using the MedCalc Software (V. 17.8; MedCalc Software, Ostend, Belgium). All data are presented as mean ± SEM and differences with *p* < 0.05 were considered statistically significant.

## Figures and Tables

**Table 1:** Semen parameters of fertile donors from control and ROS+ groups.

Parameter	WHO ^1^	Control	ROS+	*p*-Value
Volume (mL)	>1.5	4.24 ± 0.67	3.76 ± 0.72	0.4384
pH	7.6–8	7.66 ± 0.07	7.60 ± 0.05	0.3333
Sperm motility (%)	>40	55 ± 3	58 ± 6	0.5035
Sperm concentration (10^6^/mL)	>15	90.95 ± 15.59	75.02 ± 12.87	0.6221
Total sperm count (10^6^)	>39	359.50 ± 63.86	254.95 ± 49.67	0.1809
Round cells (10^6^/mL)	<1	0.82 ± 0.27	1.68 ± 0.76	0.6221
Leukocytes (10^6^/mL)	<1	0.04 ± 0.04	0.04 ± 0.04	0.9539
ROS levels (RLU/sec/10^6^ sperm)	-	46.62 ± 9.67	1053.21 ± 441.43	0.0001

Results are presented as mean ± SEM (*n* = 20). Results were considered statistically significant for *p* < 0.05. RLU, relative light units; ROS, reactive oxygen species. ^1^ World Health Organization (WHO) guidelines for human semen analysis (Low reference values, fifth centile, 95% confidence intervals).

**Table 2:** Proteomic data of the differentially expressed proteins identified in seminal plasma samples from fertile donors from control and ROS+ groups, selected for validation by Western blot.

Protein	Abundance		NSAF Ratio	Expression Profile	*p*-Value
	Control	ROS+			
SEMG1	High	High	0.26	UE in ROS+	0.00074
SEMG2	High	High	0.26	UE in ROS+	0.00023
HP	Very Low	Low	9.03	OE in ROS+	0.00349
PRDX4	Very Low	Low	3.39	OE in ROS+	0.00099
SERPINB6	Low	Low	2.68	OE in ROS+	0.00424
S100A9	Very Low	Medium	3.77	OE in ROS+	0.01707
C3	Very Low	Medium	17.22	OE in ROS+	0.00210

C3, Complement C3; HP, Haptoglobin; NSAF, Normalized spectral abundance factor; OE, overexpressed; PRDX4, Peroxiredoxin 4; S100A9, S100 calcium-binding protein A9; SEMG1, Semenogelin I; SEMG2, Semenogelin II; SERPINB6, Serpin B6; UE, underexpressed.

**Table 3:** Proteomic data of the differentially expressed proteins identified in spermatozoa samples from fertile donors from control and ROS+ groups, selected for validation by Western blot.

Protein	Abundance		NSAF Ratio	Expression Profile	*p*-Value
	Control	ROS+			
NDUFS1	Medium	Very Low	0.02	UE in ROS+	0.00004
PRDX4	Low	Medium	4.48	OE in ROS+	0.00134
SOD1	Low	Medium	3.99	OE in ROS+	0.02830
TXNRD1	-	Very Low	-	Unique to ROS+	0.00006
TXNRD2	Very Low	Medium	10.95	OE in ROS+	0.03640

NDUFS1, NADH:Ubiquinone Oxidoreductase Core Subunit S1; NSAF, Normalized spectral abundance factor; OE, overexpressed; PRDX4, Peroxiredoxin 4; SOD1, superoxide dismutase 1; TXNRD1, Thioredoxin reductase 1; TXNRD2, Thioredoxin reductase 2; UE, underexpressed.

## Supplementary Materials

The following are available online at http://www.mdpi.com/1422-0067/20/1/203/s1.
