# Supplementary material for: Proteomic Signatures Reveal Differences in Stress Response, Antioxidant Defense and Proteasomal Activity in Fertile Men with High Seminal ROS Levels
Source: Int J Mol Sci. 2019 Jan 8;20(1):203. doi: 10.3390/ijms20010203 (PMC6337289; doi:10.3390/ijms20010203)
Supplement: Supplementary file 1 [file ijms-20-00203-s001.zip › Supplementary Table 1.docx]

**Supplementary Table 1.** List of the primary and secondary antibodies used in this study.

| Antibody | Source | KDa | Dilution | Vendor | Catalog # |
| --- | --- | --- | --- | --- | --- |
| SEMG1 | Goat | 52 | 1:200 | Santa Cruz Biotechnology | sc-34719 |
| SEMG2 | Rabbit | 65 | 1:1000 | Abcam | ab108085 |
| PRDX4 | Rabbit | 27 | 1:10000 | Abcam | ab184167 |
| HP | Rabbit | 45 | 1:5000 | Abcam | ab131236 |
| SERPINB6 | Rabbit | 42 | 1:1000 | Abcam | ab97330 |
| SOD1 | Rabbit | 37-38 | 1:10000 | Abcam | ab51254 |
| NDUFS1 | Rabbit | 79 | 1:10000 | Abcam | ab157221 |
| TXNRD1 | Mouse | 55 | 1:500 | Abcam | ab16847 |
| TXNRD2 | Rabbit | 57 | 1:1000 | Abcam | ab180493 |
| S100A9 | Rabbit | 13 | 1:1000 | Abcam | ab92507 |
| C3 | Mouse | 70 | 1:1000 | Abcam | ab36989 |
| Mouse* | Rabbit | --- | 1:10000 | Abcam | ab6728 |
| Rabbit* | Goat | --- | 1:10000 | Abcam | ab97051 |
| Goat* | Donkey | --- | 1:10000 | Santa Cruz Biotechnology | sc-2020 |

*Secondary antibody. C3: Complement C3; HP: Haptoglobin; NDUFS1: NADH:Ubiquinone Oxidoreductase Core Subunit S1; PRDX4: Peroxiredoxin 4; S100A9: protein S100-A9; SEMG1: Semenogelin I; SEMG2: Semenogelin II; SERPINB6: Serpin B6; SOD1: superoxide dismutase 1; TXNRD1: Thioredoxin reductase 1; TXNRD2: Thioredoxin reductase 2.
