# Supplementary figures and images for: Proteomic Signatures Reveal Differences in Stress Response, Antioxidant Defense and Proteasomal Activity in Fertile Men with High Seminal ROS Levels
Source: Int J Mol Sci. 2019 Jan 8;20(1):203. doi: 10.3390/ijms20010203 (PMC6337289; doi:10.3390/ijms20010203)

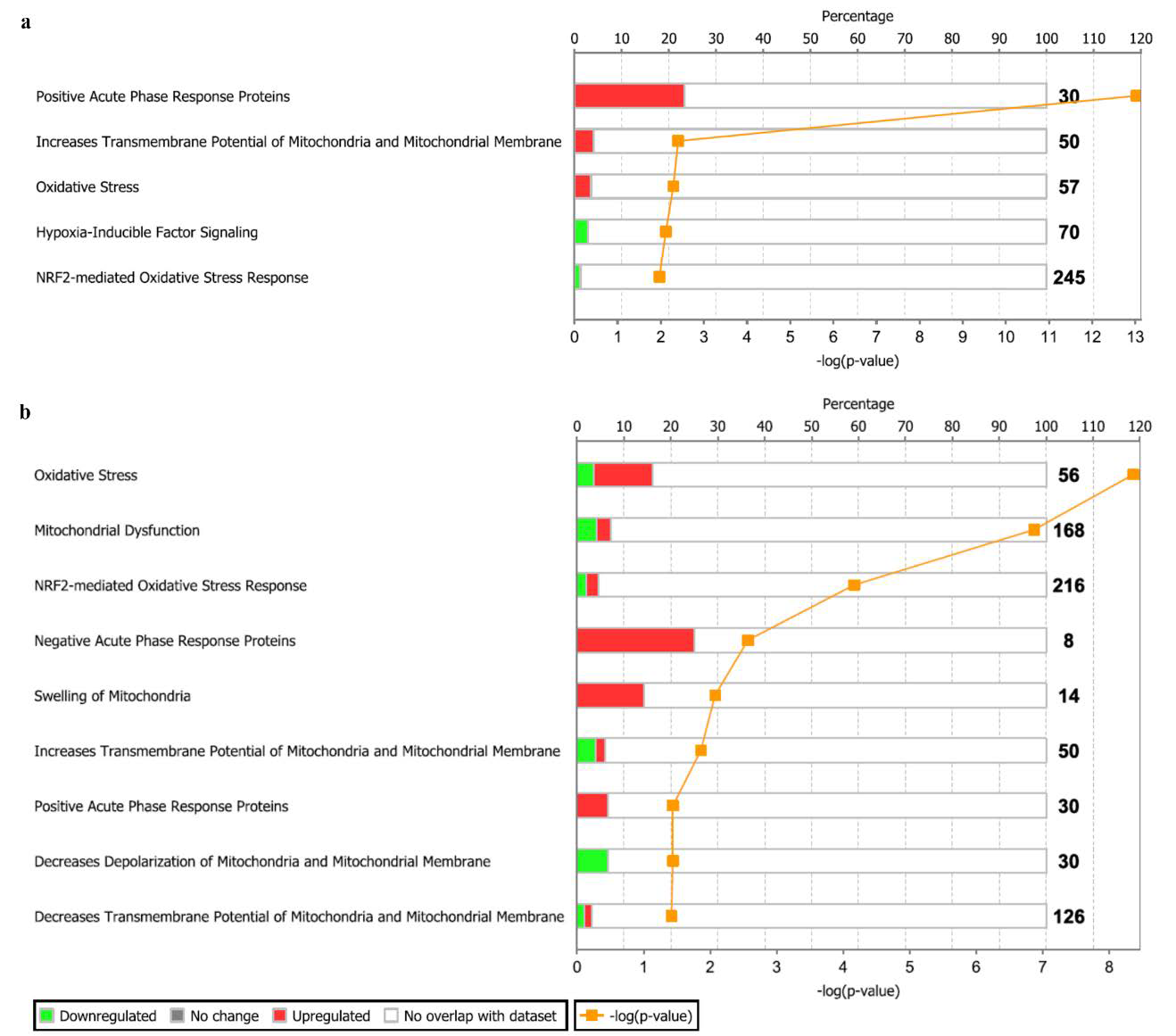

Supplement: Supplementary file 1 [file ijms-20-00203-s001.zip › Supplemental figure 1.tiff]

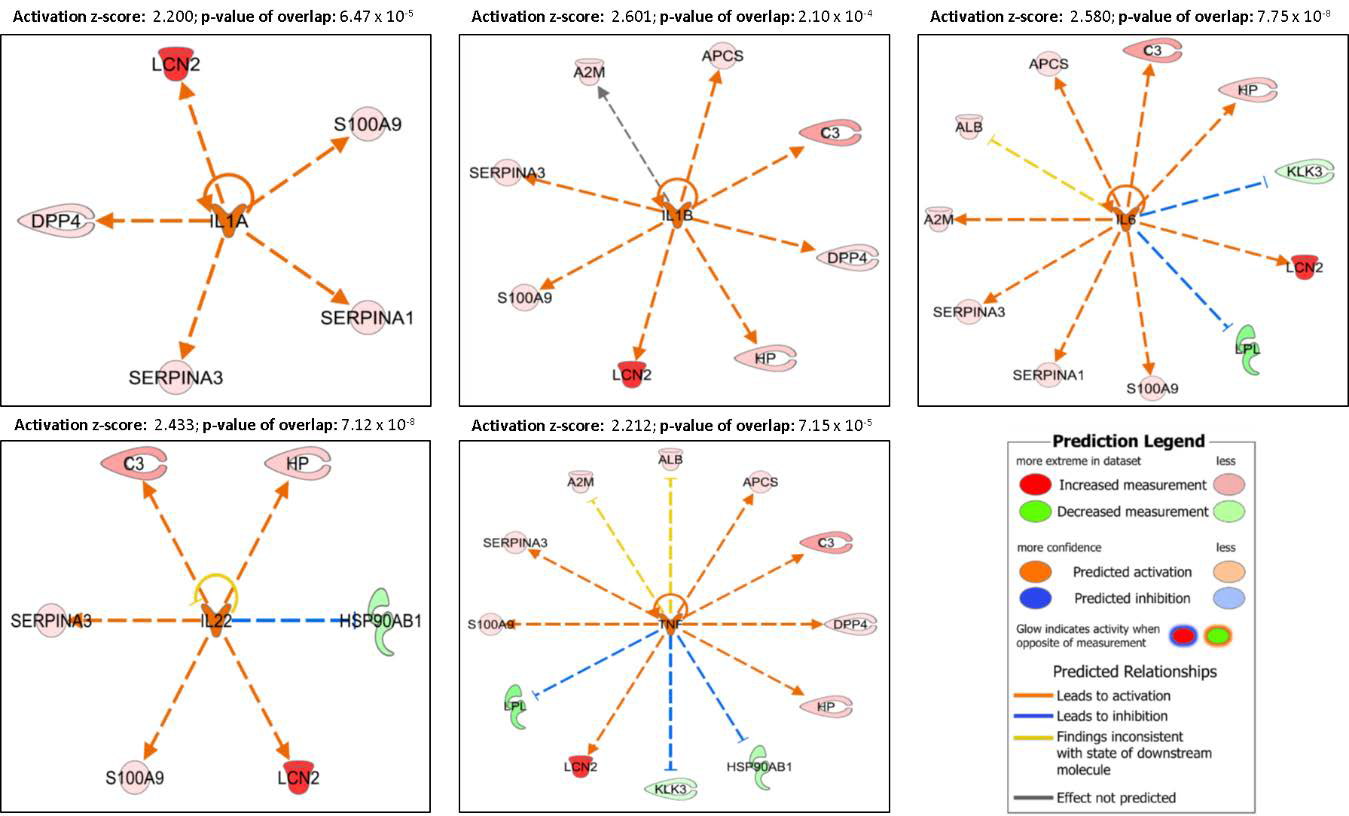

Supplement: Supplementary file 1 [file ijms-20-00203-s001.zip › Supplemental figure 2.tiff]

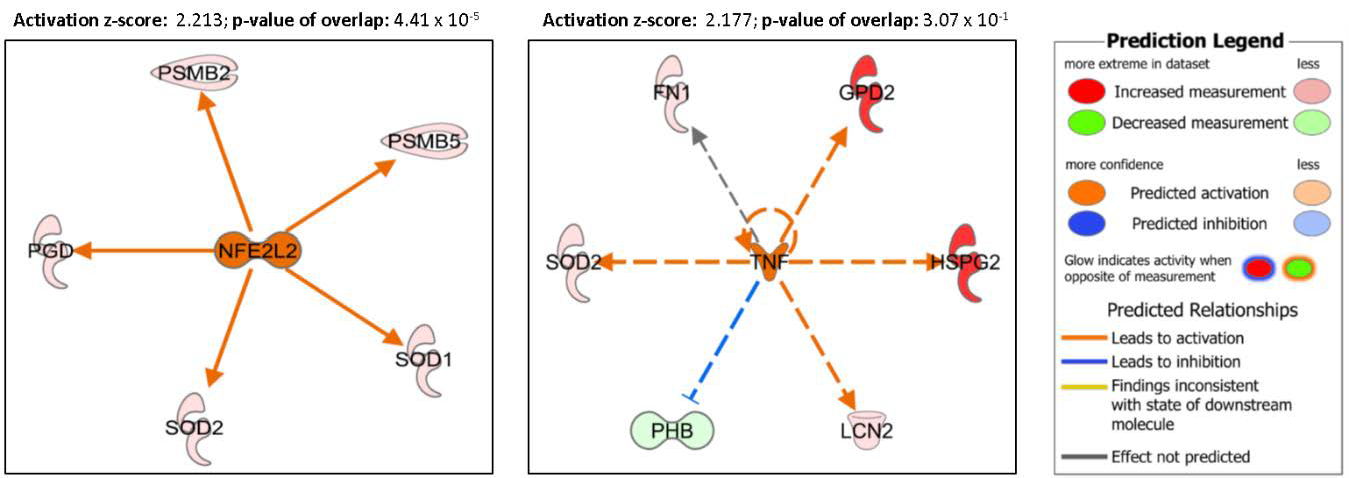

Supplement: Supplementary file 1 [file ijms-20-00203-s001.zip › Supplemental figure 3.tiff]
